# Supplementary material for: All-in-one, all-optical logic gates using liquid metal plasmon nonlinearity
Source: Nat Commun. 2024 Feb 26;15:1726. doi: 10.1038/s41467-024-46014-3 (PMC10897469; doi:10.1038/s41467-024-46014-3)
Supplement: Supplementary file 1 — Supplementary Information [file 41467_2024_46014_MOESM1_ESM.pdf]

All-in-one, all-optical logic gates using liquid metal plasmon nonlinearity

Jinlong Xu[1,2#], Chi Zhang[2#], Yulin Wang[2,3#], Mudong Wang[2], Yanming Xu[1,2], Tianqi Wei[2], Zhenda Xie[2*], Shiqiang Liu[2], Chao-Kuei Lee[4], Xiaopeng Hu[2*], Gang Zhao[2], Xinjie Lv[2], Han Zhang[5], Shining Zhu[2], Lin Zhou[2*]

[1] Department of physics, College of Physics and Information Engineering, Fuzhou University, Fuzhou, Fujian 350108, China

[2] National Laboratory of Solid State Microstructures, School of Electronic Science and Engineering, College of Engineering and Applied Sciences, School of Physics, Nanjing University, Nanjing 210093, China

[3] Department of Physics, Nanjing Tech University, Nanjing 210009, China

[4] Department of Photonics, National Sun Yat-sen University, Kaohsiung, 80424, Taiwan

[5] Key Laboratory of Optoelectronic Devices and Systems of Ministry of Education and Guangdong, College of Physics and Optoelectronic Engineering, Shenzhen University, Shenzhen, 518060, China

[#]These authors contributed equally: Jinlong Xu, Chi Zhang, Yulin Wang.

[*]Corresponding authors: xiezhenda@nju.edu.cn (Z.D.X.); xphu@nju.edu.cn (X.P.H.); linzhou@nju.edu.cn(L.Z.)

Supplementary Information

Contents

S. I Synthesis and characterization of Galinstan liquid metal nanodroplet assemblies (GNAs)

S. II Simulations of localized surface plasmon (LSP) resonances in GNAs

S. III Characterization of n_2 in a broad spectral region by spatial self-phase modulation (SSPM)

S. IV Diffraction ring pattern simulation and reversible reconfiguration between ‘0’ and ‘1’

S. V All-optical logic gates (AOLGs) operations via GNAs

S. VI Possibility of nanoscale modulation via a few GNAs

S. VII References

S. I Synthesis and characterization of Galinstan liquid metal nanodroplet assemblies (GNAs)

Galinstan alloy was synthesized via mixture and heating process. Then, liquid phase exfoliation (LPE) was used to obtain GNAs. The process was shown in **Supplementary Fig. 1**. Previous studies have proven that LPE is a facile and efficient technique to produce pure nanoscale materials for investigating their intrinsic properties, because it can avoid the impurity and contamination problems commonly seen in chemical synthesis. Compared with a variety of common hypotoxic solvents, such as alcohol, acetone and dichloromethane, we found that sonication of Galinstan in N-methyl-2-pyrrolidinone (NMP) resulted in a much higher yield and more steady suspensions. It may be attributed to the matched surface energy between NMP and GNAs. Via transmission electron microscope (TEM) characterization, the collective

diameters of the GNAs have the approximately normal distribution within 30-150 nm. Energy dispersive X-ray spectrum (EDS) measurement confirmed the element components of Ga:In:Sn is 63:24:13 in the GNAs, in consistent with the ingredient ratio, as shown in **Supplementary Fig. 3**.

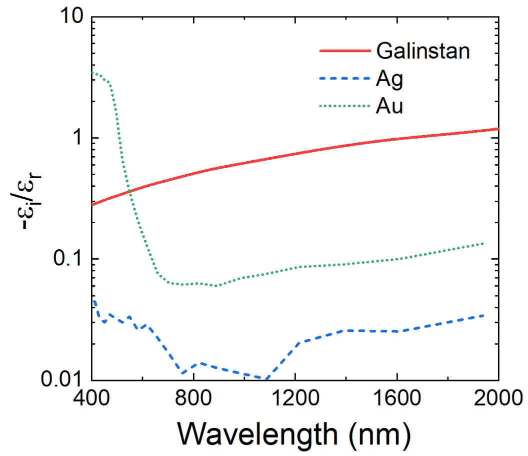

Supplementary Figure 1. Figure of merit ($-\varepsilon_i / \varepsilon_r$) for plasmonic absorption of bulk Galinstan liquid.

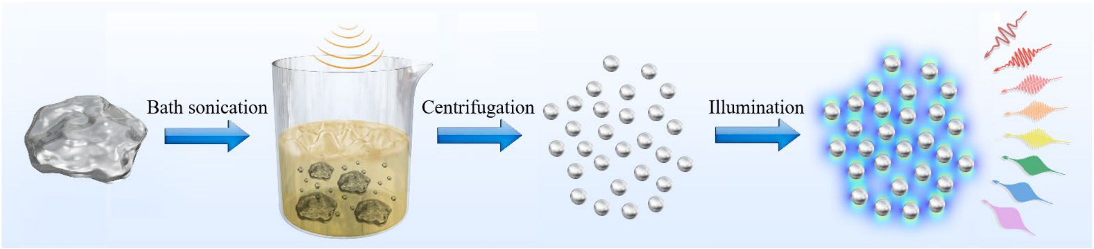

Supplementary Figure 2. Synthesis process of GNAs.

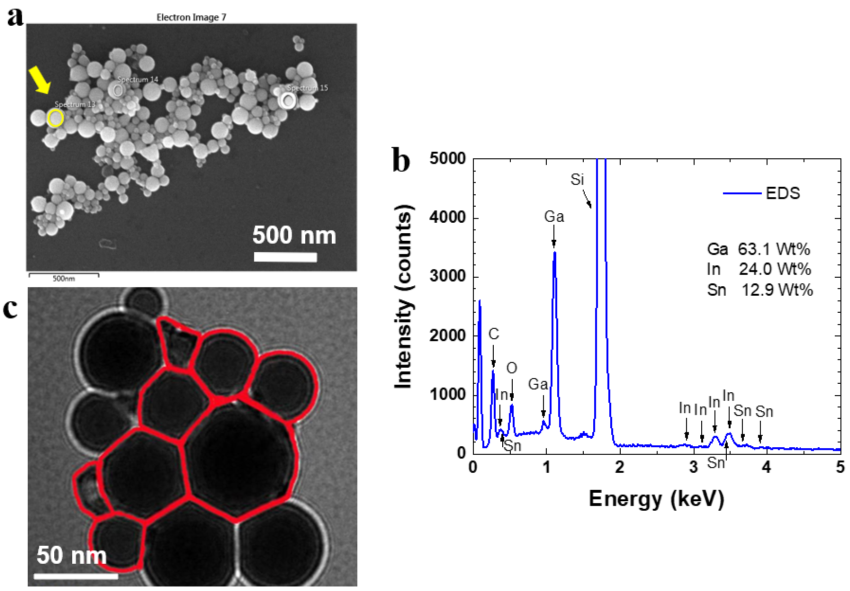

Supplementary Figure 3. Characterization of GNAs. (a,b) SEM graph of GNAs and corresponding EDS spectrum at the arrow section. (c) GNAs morphed into massive irregular geometries in TEM characterization.

S. II Simulations of localized surface plasmon (LSP) resonances in GNAs

FDTD simulations were carried out to show the LSP of typical nanodroplets in the GNAs. The dielectric constant of Galinstan nanodroplets was measured by ellipsometer and used for modeling. The surrounding medium was set to be NMP with refractive index of 1.47. Nanodroplets with diameters of 20, 40, 100, 200 nm were set in the simulation region. The wavelength of the optical source was set to be 210-2500 nm. **Supplementary Fig. 4** shows the electric field near the isolated nanodroplets at several typical wavelengths. It can be clearly seen that, 20 and 40 nm nanodroplets have stronger LSP at UV excitation. While at visible region, nanodroplets with diameter of 100 nm have stronger LSP. At infrared region, LSP around 200 nm nanodroplets is stronger. This size dependent LSP is one of the important factors to broadband absorption of GNAs.

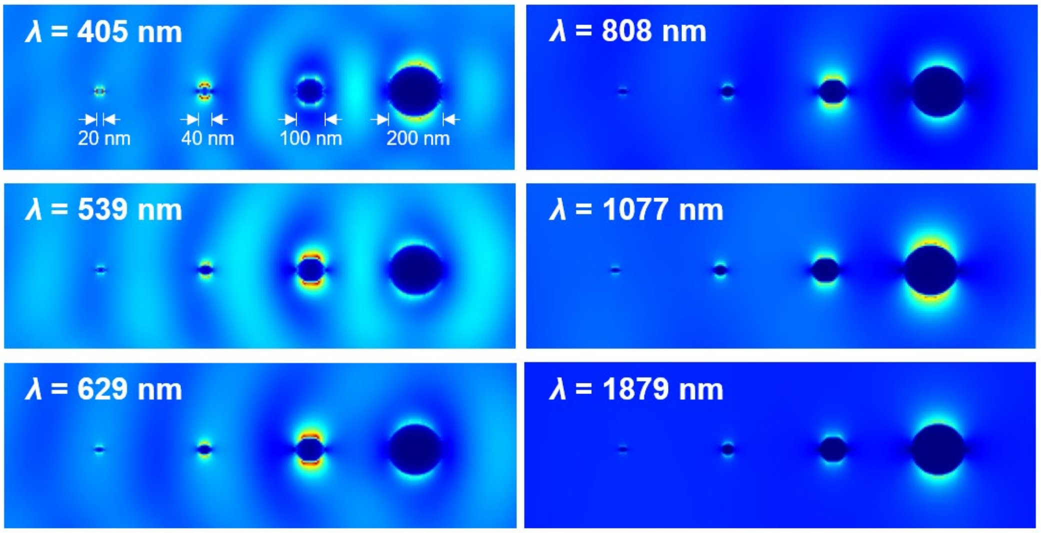

Supplementary Figure 4. FDTD simulations of LSP field distribution depending on the size of isolated Galinstan nanodroplets.

Besides, the LSP frequency is highly hybridized due to the unique advantages of GNAs such as wide-distribution nanodroplet (or nanoparticles) size, excellent inherent stretchability of liquid phase, and strong resonant interparticle coupling. Their combination provides a high density and numerous kinds of hybrid optical modes. The corresponding LSP frequency changes largely for different combinations. This is further analyzed based on simulations with six different situations, including single nanodroplet sphere with a diameter of 100 nm, single nanodroplet ellipse morphed from the sphere above with stretched by 1.16 times in one axis (namely three major axes of 116/92/92 nm), two spheres with gap of 31 nm, two ellipses morphed in situ with gap of 1 nm, and three/four morphed ellipses with gap of 1nm, as depicted in **Supplementary Fig. 5a**. According to the simulation results, single nanodroplet absorption is generally weaker than multi-nanodroplets with strong gap coupling. As the number of nanodroplets increases, the resonance peak exhibits red shift and the absorption becomes stronger. Moreover, when two ellipses morph in situ, the nanodroplets get closer (gap changes from 31 to 1 nm), the coupling among nanodroplets gets stronger leading to stronger absorption and red shift in spectrum. The corresponding electric field distributions for the six situations at resonance peak are shown in **Supplementary Fig. 5b-g.** In the experiment, there are usually

numerous GNAs with random deformation and coupling as shown in **Supplementary Fig. 3**, which can result in strong broadband absorption.

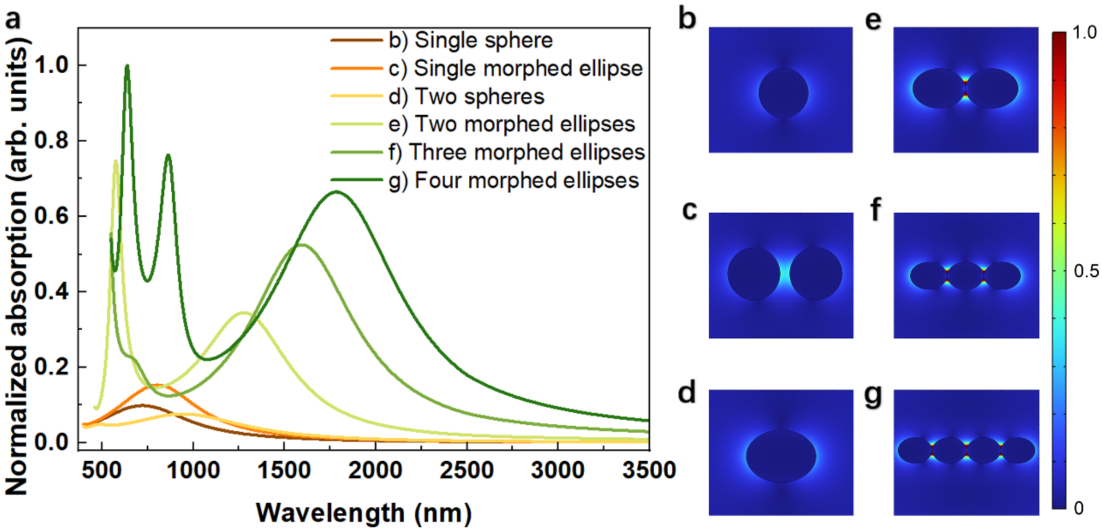

Supplementary Figure 5. Simulation of LSP frequency depending on the stretchability and interparticle coupling of Galinstan nanodroplets. (a) LSP absorption spectra and (b-g) electric field distributions for six typical situations of single or few nanodroplets with different stretchability and interparticle coupling.

As mentioned above, the high density of plasmonic modes stemmed from the wide-distribution sizes and flexible stretchability of GNAs leads to strong confinement of LSP field with an ultrabroad band across visual to infrared regions. This effect can be further verified by simulating the LSP field of randomly distributed GNAs as delineated in **Supplementary Fig. 6a.** One can see that many hot spots of LSP with strong field confinement can generate stably under wideband excitation. The change of hot-spot position with excitation wavelength among different gaps of adjacent GNAs evidences the hybridization of different plasmonic modes responsive to different excitation wavelengths. Benefiting from this field confinement, as indicated in **Supplementary Fig. 6b**, the optical field of excitation light at the spectral region of 400-4000 nm can be strongly enhanced by 10-16 times, contributing to the ultrabroadband thermo-optical nonlinearity of GNAs.

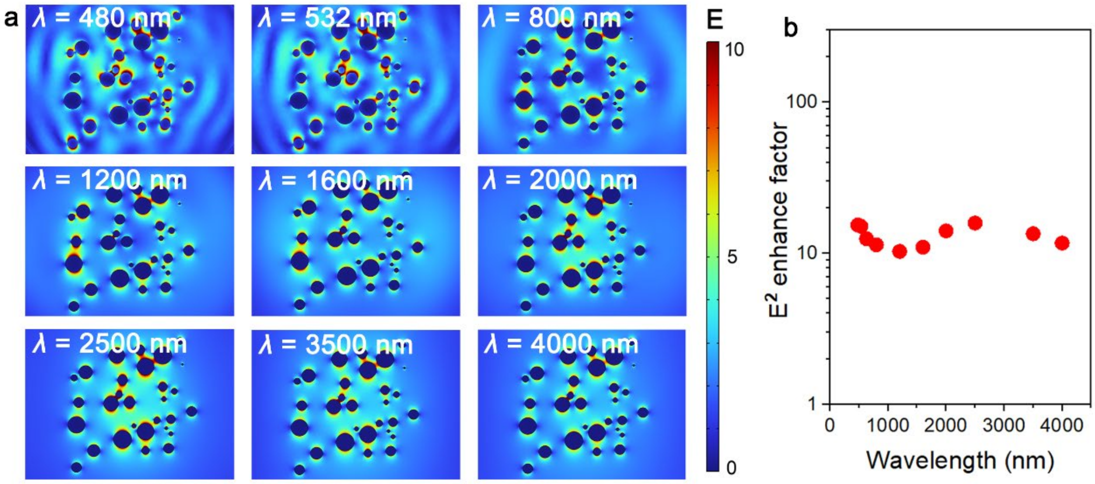

Supplementary Figure 6. (a) Simulations of broadband LSP in random distributed GNAs. The diameters of nanodroplets are set to be 20-160 nm. (b) Corresponding enhance factor calculated by integrating the LSP field excited at different wavelengths in (a).

S. III Characterization of n_2 in a broad spectral region by spatial self-phase modulation (SSPM)

The setup for the SSPM experiment is illustrated as **Supplementary Fig. 7**. The GNA suspensions were contained in a commercial JGS1 quartz cuvette with an optical length of 1 mm. The excited light sources were three continuous wave (CW) lasers at the wavelength of 405, 532 and 1064 nm and one femtosecond-pulsed laser at the wavelength of 776 nm, all focused into the cuvette by a convex lens. In this case, the intensity-dependent refractive index $n = n_0 + n_2 I(r,z)$ of the GNA suspensions leads to a nonlinear phase shift $\Delta\phi(r)$, which can be expressed by

$$\Delta\phi(\mathrm{r}) = \left(\frac{2\pi n_0}{\lambda}\right)\int_0^L n_2 I(r,z)\,dz \qquad \text{(SE1)}$$

where n_0 and n_2 are the linear and effective-nonlinear refractive index, respectively; r is the radial coordinate; λ is the laser wavelength; L is the effective optical length of the sample; and $I(r, z)$ is the distribution of the incident light intensity with z as the axial coordinate along the light propagation.

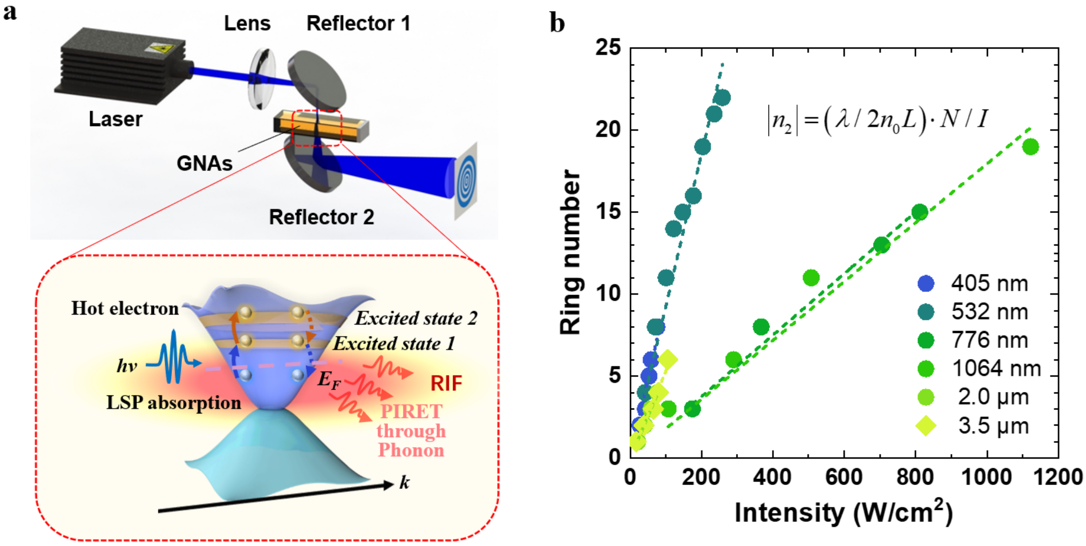

Supplementary Figure 7. (a) Mechanism and (b) measurement of the optical nonlinearity of Galinstan suspensions.

For a typical Gaussian beam, the $I(r)$ is proportional to the factor exp($-2r^2/a^2$) and so as the $\Delta\phi(r)$, where a is the beam radius. Transverse wave vector $k_r = d\phi / dr$ decided the beam angle, and the beams with the same direction will interfere at far field. Constructive interference occurs when the phase difference $|\Delta\phi(0) - \Delta\phi(\infty)|$ $\approx(2N\text{-}1)\pi$ (N = 1, 2, 3…) with the appearance of bright ring referred to as Nth-order diffraction ring mode (N is also the ring number). If $|\Delta\phi(0) - \Delta\phi(\infty)|$ is much larger than π, then a series of concentric interference rings appear. Through the simple derivation from **Equation (SE1)**, we obtained the dependence of the ring number upon the incident light as

$$N \approx \frac{2n_0 |n_2| L}{\lambda} I \tag{SE2}$$

where I is the average intensity of the incident light, which is approximately half of the value of $I(0, z)$. The monotonous relation of N and I fits well with our experiments (Fig. 2c). Therefore, the effective nonlinearity n_2 for different wavelengths can be extracted by fitting **Equation (SE2)** to the experimental data in Fig. 2c. The nonlinear refractive index n_2 of GNAs was derived to be 10^{-5} cm^2/W via $|n_2| = (\lambda / 2n_0 L) \cdot N / I$,

where λ is the vacuum wavelength, n_0 is the refractive index in weak field, and L is the propagation length. The n_2 of Galinstan is higher than most nanomaterials reported including graphene, $CsPbI_3$ et al., as shown in **Table S1**.

Table S1. Comparison for nonlinear refractive index n_2 of typical nanomaterials

Materials	Type of laser	n_2 (cm²/W)	Ref.
Graphene	CW 633 nm	1.13×10^{-5}	1
MoS_2	CW 488 nm	9.32×10^{-7}	2
Black Phosphorus	350/600/700/750/1160 nm	10^{-5}	3
SnS	532/633 nm	$2.317/0.323 \times 10^{-5}$	4
Bismuthene	CW 532/633 nm & Pulse 400/800 nm	10^{-6}	5
Antimonene	CW 532/633 nm	$2.88/0.979 \times 10^{-5}$	6
$CsPbI_3$	CW 457/532 nm	$2.56/0.578 \times 10^{-5}$	7
Bi_2Se_3	Pulse 350/600/700/1160 nm	$1.16/0.35/0.25/0.165 \times 10^{-8}$	8
Bi_2Te_3	Pulse 1070 nm	2.11×10^{-8}	9
$Ti_3C_2T_x$	CW 457/532/671 nm	$11.011/4.725/0.447 \times 10^{-4}$	10
$nTaSe_2$	CW 532/671 nm	$8.0/3.3 \times 10^{-7}$	11
Graphene oxide	CW 532/671 nm	$3.57/1.1 \times 10^{-6}$	12
Galinstan	CW 405 nm<br>CW 532 nm<br>CW 766 nm<br>CW 1064 nm<br>CW 2000 nm<br>CW 3500 nm	1.33×10^{-5}<br>1.68×10^{-5}<br>4.95×10^{-6}<br>6.5×10^{-6}<br>3.48×10^{-5}<br>5.95×10^{-5}	This work

n_2: effective-nonlinear refractive index

S. IV Diffraction ring pattern simulation and reversible reconfiguration between ‘0’ and ‘1’

According to Kirchhoff’ s diffraction formula[13], the far-field pattern is decided by

$$I\left(r,z_0\right)=\left(\frac{2\pi}{\lambda z_0}\right)^2 I_0\left|\int_0^\infty J_0\left(\frac{2\pi r r_1}{\lambda z_0}\right)\exp\left(\frac{-r_1^2}{w\left(z_1\right)^2}\right)\exp\left[-i\left(\varphi_{\mathrm{L}}+\varphi_{\mathrm{NL}}\right)\right]r_1 dr_1\right|^2 \quad \text{(SE3)}$$

where r and r_1 are the radius coordinates on the observation plane and at the GNA

dispersion, respectively; z_0 is the distance between the sample and the observation plane; z_1 is the position of the sample on the optical axis away from the focal point of the lens; $w = w_0\sqrt{1+\left[\lambda z_1/\left(\pi w_0^2\right)\right]^2}$ is the beam waist at the sample; w_0 is the beam waist at the focal point; $\varphi_{\mathrm{L}} = k\left[r_1^2/\left(2z_0\right)+r_1^2/\left(2R\right)\right]$ is the linear phase shift; $\varphi_{\mathrm{NL}} = kn_2 I\, w_0^2/w^2\, L\exp\left(-r_1^2/w^2\right)$ is the nonlinear phase shift; $R = z_1\left\{1+\left[\pi w_0^2/\left(\lambda z_1\right)\right]^2\right\}$ is the curvature radius of the wave front at the position of the sample; and J_0 is the zeroth-order Bessel function. When $R \ll Z_0$, which is consistent with the experiment, $\varphi_{\mathrm{L}} = k\,r_1^2/\left(2R\right) = k\,r_1^2\Big/\left\{2z_1\left[1+\left(\pi w_0^2/\left(\lambda z_1\right)\right)^2\right]\right\}$. Particularly, if $\pi w_0^2/\lambda \ll \sqrt{z_1 z_0}$ and $z_1 \ll \frac{\pi w_0^2}{\lambda}$, φ_L will be linear with z_1.

To realize all-optical logic gate, we defined ‘bright center’ (fundamental Gaussian beam) and ‘dark center’ (1-order diffraction ring mode) at the center of the far-field pattern as logic ‘1’ and ‘0’. Under the assumption of z_0 = 200 mm, λ = 532 nm, waist radius w_0 = 0.021 mm, the $\Delta\varphi_{\mathrm{L}}(r1)$ and $\Delta\varphi_{\mathrm{NL}}(r1)$ at different z_1 are calculated (**Supplementary Fig. 8**). We define the final phase-shift $\Delta\varphi = \Delta\varphi_{\mathrm{L}} + \Delta\varphi_{\mathrm{NL}}$ in the beam diameter, and it is clear that ‘1’ and ‘0’ can be implied by the relationship between $\Delta\varphi$ and π. Far-field pattern under similar condition (z_0 = 200 mm, z_1=0.001 mm, λ = 532 nm, waist radius w_0 = 0.034 mm) at different $\Delta\varphi$ was calculated from Equation 1.

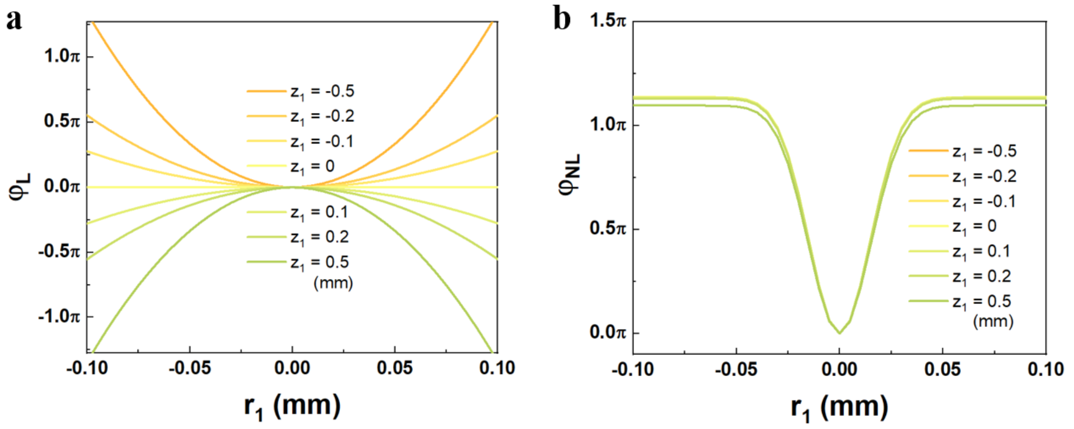

Supplementary Figure 8. Dependence of (a) linear phase-shift φ_L and (b) nonlinear phase-shift φ_{NL} on radius coordinates of laser beam modulated by the nonlinear RIF of GNAs. The calculation is performed under the assumption of z_0 = 200 mm, λ = 532 nm, waist radius w_0 = 0.021 mm. $\Delta\varphi_{NL}$ (r_1 = ±0.10 mm) at z_1 = 0 mm is set to be -π.

S. V All-optical logic gates (AOLGs) operations via GNAs

For the 'IMP' gate, the focal position of control beam is in front of the focal position of signal beam, and the sign of $\Delta\varphi_L$ and $\Delta\varphi_{NL}$ are opposite. When signal beam was '1' and control beam was '1', the nonlinear phase-shift ($\Delta\varphi_{NL}$ > 0) from control beam maintained the $|\Delta\varphi|$ to smaller than π, leading to output '1'. When signal beam was '1' and control beam was '0', the nonlinear phase-shift ($\Delta\varphi_{NL}$ > 0) from control beam maintained the $|\Delta\varphi|$ to smaller than π, leading to output '1'. When signal beam was '0' and control beam was '1', the nonlinear phase-shift from control beam '1' maintained the $|\Delta\varphi|$ to larger than π, leading to output '0'. When signal beam was '0' and control beam was '0', the nonlinear phase-shift from control beam '1' decreased the $|\Delta\varphi|$ to smaller than π, leading to output '1'. The repeatable switching operation of this 'IMP' gate in 100 seconds was carried out. Experiments at

1342 nm and 2 μm were also demonstrated to access 'IMP' gate, implying the broad operation bandwidth of Galinstan ALOGs over VIS to NIR. The contrast between the central intensity of '1' and '0' patterns is within the range of 2-10, enough for precise identification of 1' and '0' states. These results were shown in **Fig. 4b**.

For the 'OR' gate, the focal position of control beam is in front of the focal position of signal beam, and the sign of $\Delta\varphi_L$ and $\Delta\varphi_{NL}$ are opposite. When signal beam was '1' and control beam was '1', the nonlinear phase-shift ($\Delta\varphi_{NL} > 0$) from control beam was too weak to increase the $|\Delta\varphi|$ to π, leading to output '1'. When signal beam was '1' and control beam was '0', the nonlinear phase-shift ($\Delta\varphi_{NL} > 0$) from control beam was also too weak to increase the $|\Delta\varphi|$ to π, leading to output '1'. When signal beam was '0' and control beam was '1', the nonlinear phase-shift from control beam '1' decreased the $|\Delta\varphi|$ to smaller than π, leading to output '1'. When signal beam was '0' and control beam was '0', the nonlinear phase-shift from control beam '1' can not decrease the $|\Delta\varphi|$ to smaller than π, leading to output '0'. The repeatable switching operation of this 'OR' gate in 100 secoGNAs was carried out. Experiments at 1342 nm and 2 μm were also demonstrated to access 'OR' gate, implying the broad operation bandwidth of Galinstan ALOGs over visible to infrared region. These results are shown in **Supplementary Fig. 9**.

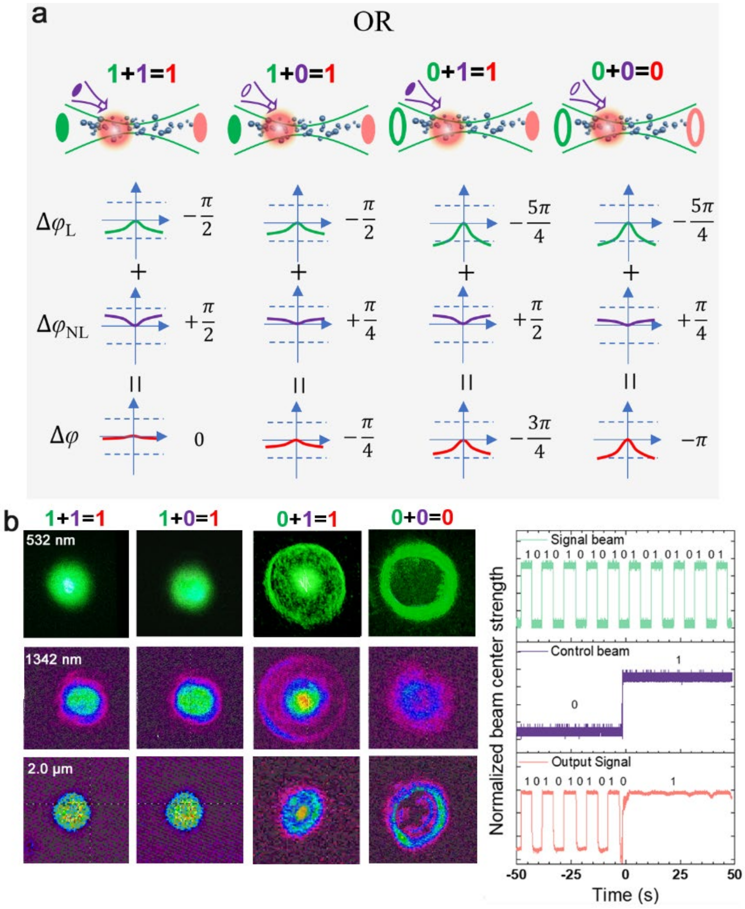

Supplementary Figure 9. Design and experimental results of OR gate. (a) Mechanism of phase modulation and corresponding scheme of phase dependence. (b) Left: spatial output patterns of OR gate across visible and infrared region. Right: temporal input and output in processing.

For the 'NOT' gate, the focal position of control beam is in front of the focal position of signal beam, and the sign of $\Delta\varphi_L$ and $\Delta\varphi_{NL}$ are opposite. Control beam was fixed to be '0'. When signal beam was '1', the nonlinear phase-shift ($\Delta\varphi_{NL} > 0$) from control beam was strong enough to increase the $|\Delta\varphi|$ to π, leading to output '0'. When signal beam was '0', the nonlinear phase-shift ($\Delta\varphi_{NL} > 0$) from control beam was decrease the $|\Delta\varphi|$ to smaller than π, leading to output '1'. The repeatable switching operation of this 'NOT' gate in 100 secoGNAs was carried out. Experiments at 1342 nm and 2 μm were also demonstrated to access 'NOT' gate,

implying the broad operation bandwidth of Galinstan ALOGs over visible to infrared region. These results are shown in **Supplementary Fig. 10**.

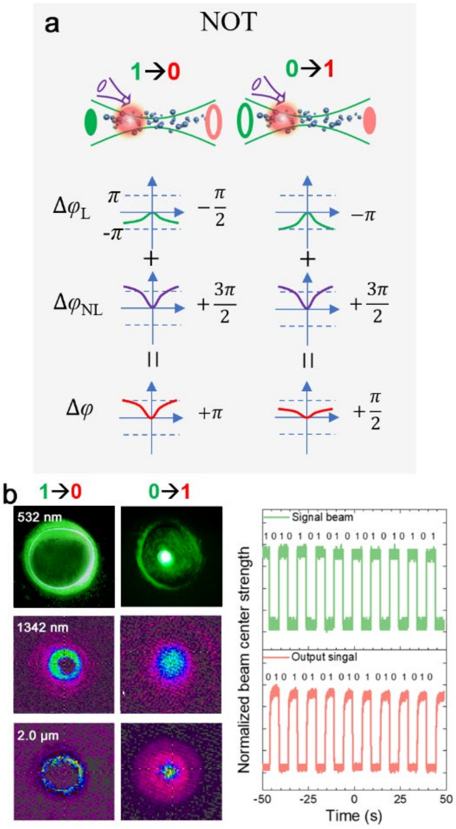

Supplementary Figure 10. Design and experimental results of 'NOT' gate. (a) Mechanism of phase modulation and corresponding scheme of phase dependence. (b) Left: spatial output patterns of NOT gate across visible and infrared region. Right: temporal input and output in processing.

For the 'NAND' gate, the focal position of control beam is in front of the focal position of signal beam, and the sign of $\Delta\varphi_{\mathrm{L}}$ and $\Delta\varphi_{NL}$ are opposite. When signal beam was '1' and control beam was '1', the nonlinear phase-shift ($\Delta\varphi_{NL} > 0$) from control beam increased the $|\Delta\varphi|$ to π, leading to output '0'. When signal beam was '1' and control beam was '0', the nonlinear phase-shift ($\Delta\varphi_{NL} > 0$) from control beam can not increase the $|\Delta\varphi|$ to π, leading to output '1'. When signal beam was '0' and

control beam was ‘1’, the nonlinear phase-shift from control beam ‘1’ decreased the $|\Delta\varphi|$ to smaller than π, leading to output ‘1’. When signal beam was ‘0’ and control beam was ‘0’, the nonlinear phase-shift from control beam ‘1’ decreased the $|\Delta\varphi|$ to smaller than π, leading to output ‘1’. The repeatable switching operation of this ‘NAND’ gate in 100 secoGNAs was carried out. Experiments at 1342 nm and 2 μm were also demonstrated to access ‘NAND’ gate, implying the broad operation bandwidth of Galinstan ALOGs over visible to infrared region. These results are shown in **Supplementary Fig. 11**.

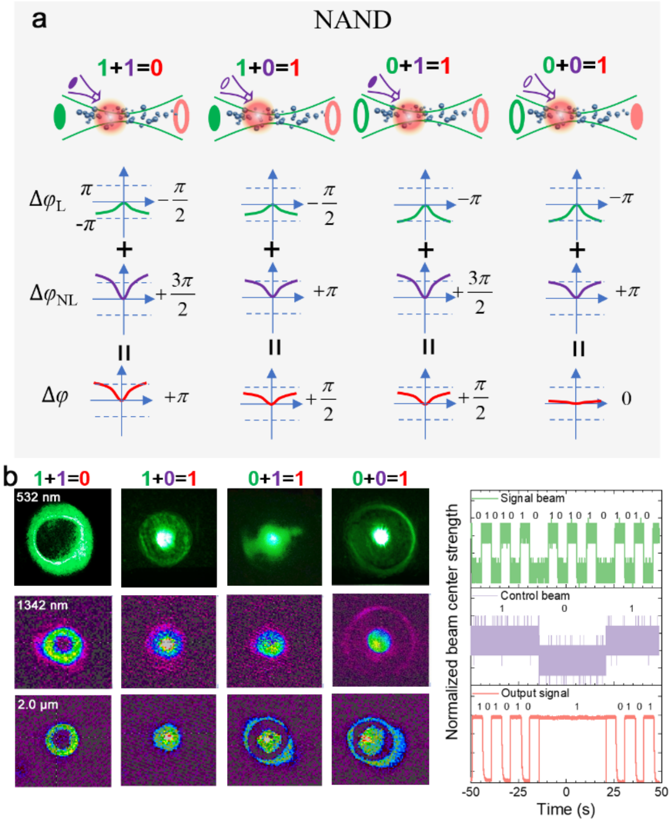

Supplementary Figure 11. Design and experimental results of ‘NAND’ gate. (a) Mechanism of phase modulation and corresponding scheme of phase dependence. (b) Left: spatial output patterns of NAND gate across visible and infrared region. Right: temporal input and output in processing.

For the ‘NOR’ gate, the focal position of control beam is in front of the focal

position of signal beam, and the sign of $\Delta\varphi_L$ and $\Delta\varphi_{NL}$ are opposite. When signal beam was '1' and control beam was '1', the nonlinear phase-shift ($\Delta\varphi_{NL}$ > 0) from control beam increased the $|\Delta\varphi|$ to π, leading to output '0'. When signal beam was '1' and control beam was '0', the nonlinear phase-shift ($\Delta\varphi_{NL}$ > 0) from control beam increased the $|\Delta\varphi|$ to π, leading to output '0'. When signal beam was '0' and control beam was '1', the nonlinear phase-shift from control beam '1' made the $|\Delta\varphi|$ to π, leading to output '0'. When signal beam was '0' and control beam was '0', the nonlinear phase-shift from control beam '1' decreased the $|\Delta\varphi|$ to smaller than π, leading to output '1'. The repeatable switching operation of this 'NOR' gate in 100 secoGNAs was carried out. Experiments at 1342 nm and 2 μm were also demonstrated to access 'NOR' gate, implying the broad operation bandwidth of Galinstan ALOGs over visible to infrared region. These results are shown in **Supplementary Fig. 12**.

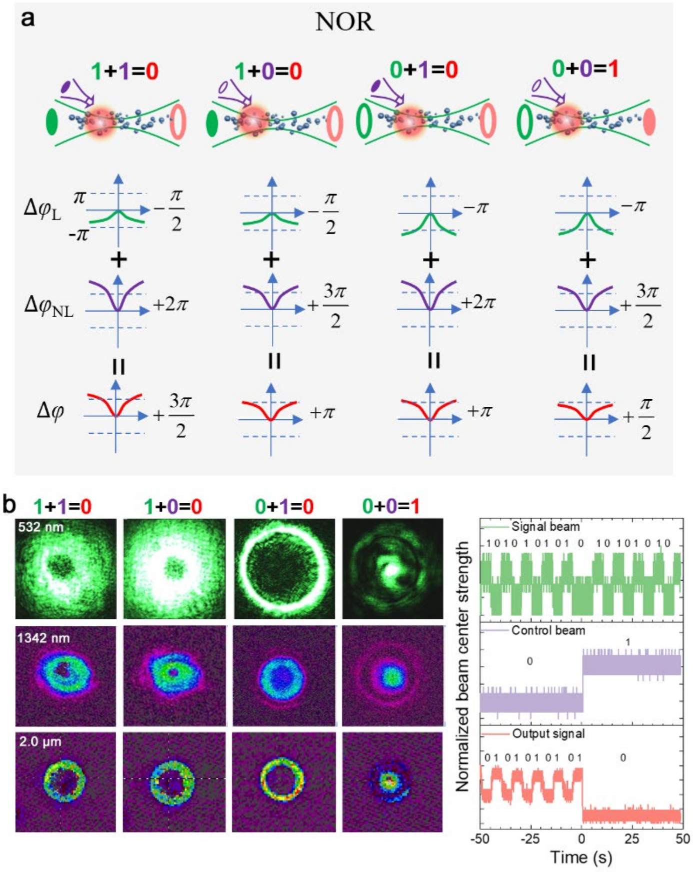

Supplementary Figure 12. Design and experimental results of 'NOR' gate. (a) Mechanism of phase modulation and corresponding scheme of phase dependence. (b) Left: spatial output patterns of NOR gate across visible and infrared region. Right: temporal input and output in processing.

For the 'XNOR' gate, the focal position of control beam is in front of the focal position of signal beam, and the sign of $\Delta\varphi_L$ and $\Delta\varphi_{NL}$ are opposite. When signal beam was '1' and control beam was '1', the nonlinear phase-shift ($\Delta\varphi_{NL} > 0$) from control beam maintained the $|\Delta\varphi|$ to smaller than π, leading to output '1'. When signal beam was '1' and control beam was '0', the nonlinear phase-shift ($\Delta\varphi_{NL} > 0$) from control beam increased the $|\Delta\varphi|$ to π, leading to output '0'. When signal beam was '0' and control beam was '1', the nonlinear phase-shift from control beam '1' maintained the $|\Delta\varphi|$ to larger than π, leading to output '0'. When signal beam was '0'

and control beam was ‘0’, the nonlinear phase-shift from control beam ‘1’ decreased the $|\Delta\varphi|$ to smaller than π, leading to output ‘1’. The repeatable switching operation of this ‘XNOR’ gate in 100 secoGNAs was carried out. Experiments at 1342 nm and 2 μm were also demonstrated to access ‘XNOR’ gate, implying the broad operation bandwidth of Galinstan ALOGs over visible to infrared region. These results are shown in **Supplementary Fig. 13**.

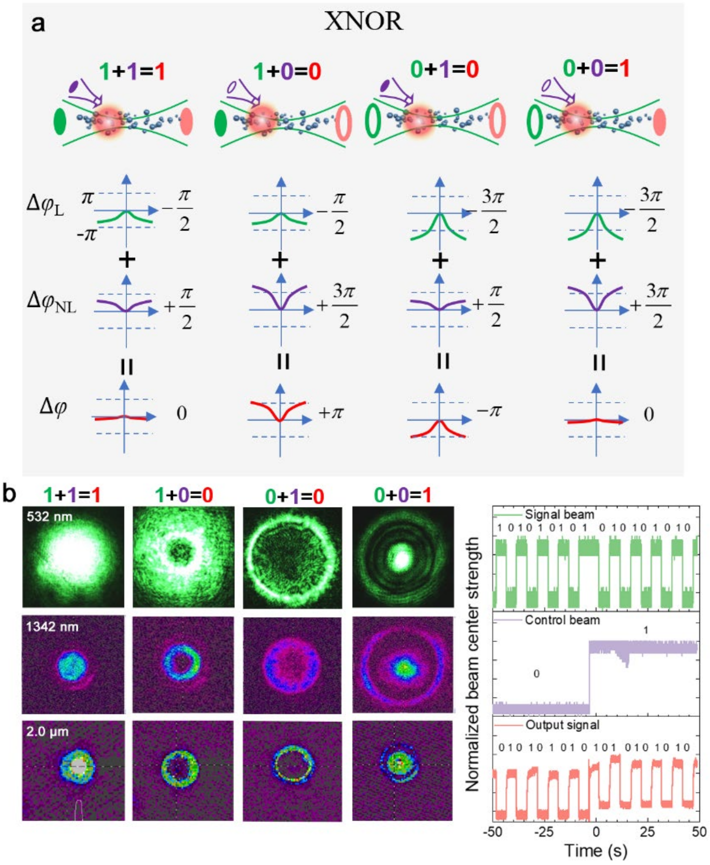

Supplementary Figure 13. Design and experimental results of ‘XNOR’ gate. (a) Mechanism of phase modulation and corresponding scheme of phase dependence. (b) Left: spatial output patterns of XNOR gate across visible and infrared region. Right: temporal input and output in processing.

For the ‘XOR’ gate, the focal position of control beam is in front of the focal position of signal beam, and the sign of $\Delta\varphi_L$ and $\Delta\varphi_{NL}$ are opposite. When signal

beam was '1' and control beam was '1', the nonlinear phase-shift ($\Delta\varphi_{NL}$ > 0) from control beam increased the $|\Delta\varphi|$ to π, leading to output '0'. When signal beam was '1' and control beam was '0', the nonlinear phase-shift ($\Delta\varphi_{NL}$ > 0) from control beam maintained the $|\Delta\varphi|$ to smaller than π, leading to output '1'. When signal beam was '0' and control beam was '1', the nonlinear phase-shift from control beam '1' decreased the $|\Delta\varphi|$ to smaller than π, leading to output '1'. When signal beam was '0' and control beam was '0', the nonlinear phase-shift from control beam '1' maintained the $|\Delta\varphi|$ to larger than π, leading to output '0'. The repeatable switching operation of this 'XOR' gate in 100 secoGNAs was carried out. Experiments at 1342 nm and 2 μm were also demonstrated to access 'XOR' gate, implying the broad operation bandwidth of Galinstan ALOGs over visible to infrared region. These results are shown in **Supplementary Fig. 14**.

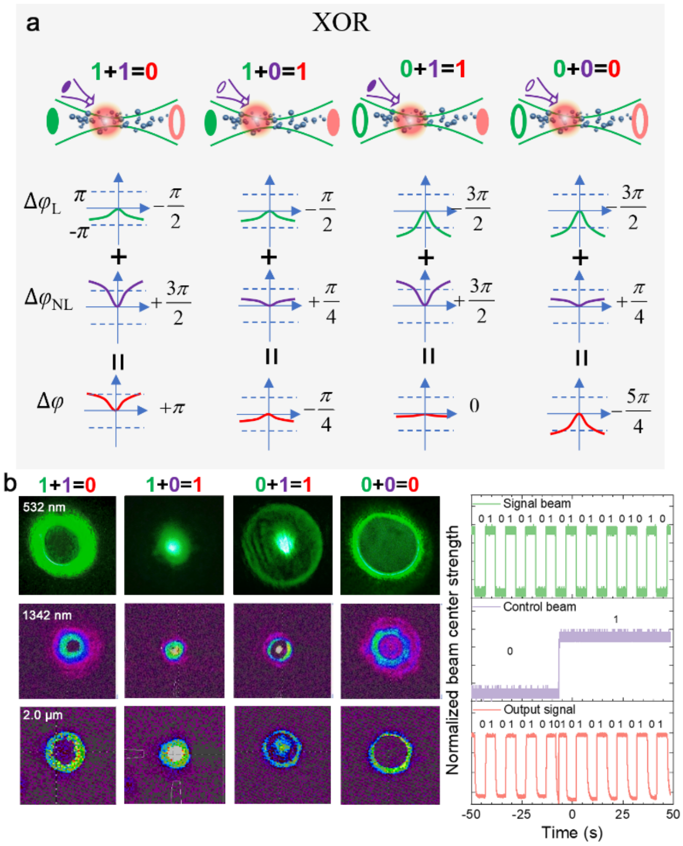

Supplementary Figure 14. Design and experimental results of ‘XOR’ gate. (a) Mechanism of phase modulation and corresponding scheme of phase dependence. (b) Left: spatial output patterns of XOR gate across visible and infrared region. Right: temporal input and output in processing.

For the ‘NIMP’ gate, the focal position of control beam is in front of the focal position of signal beam, and the sign of $\Delta\varphi_L$ and $\Delta\varphi_{NL}$ are opposite. When signal beam was ‘1’ and control beam was ‘1’, the nonlinear phase-shift ($\Delta\varphi_{NL}$ > 0) from control beam increased the $|\Delta\varphi|$ to π, leading to output ‘0’. When signal beam was ‘1’ and control beam was ‘0’, the nonlinear phase-shift ($\Delta\varphi_{NL}$ > 0) from control beam maintained the $|\Delta\varphi|$ to smaller than π, leading to output ‘1’. When signal beam was ‘0’ and control beam was ‘1’, the nonlinear phase-shift from control beam ‘1’ maintained the $|\Delta\varphi|$ to larger than π, leading to output ‘0’. When signal beam was ‘0’ and control

beam was ‘0’, the nonlinear phase-shift from control beam ‘1’ maintained the $|\Delta\varphi|$ to larger than π, leading to output ‘0’. The repeatable switching operation of this ‘NIMP’ gate in 100 secoGNAs was carried out. Experiments at 1342 nm was also demonstrated to access ‘NIMP’ gate, implying the broad operation bandwidth of Galinstan ALOGs over visible to infrared region. These results are shown in **Supplementary Fig. 15**.

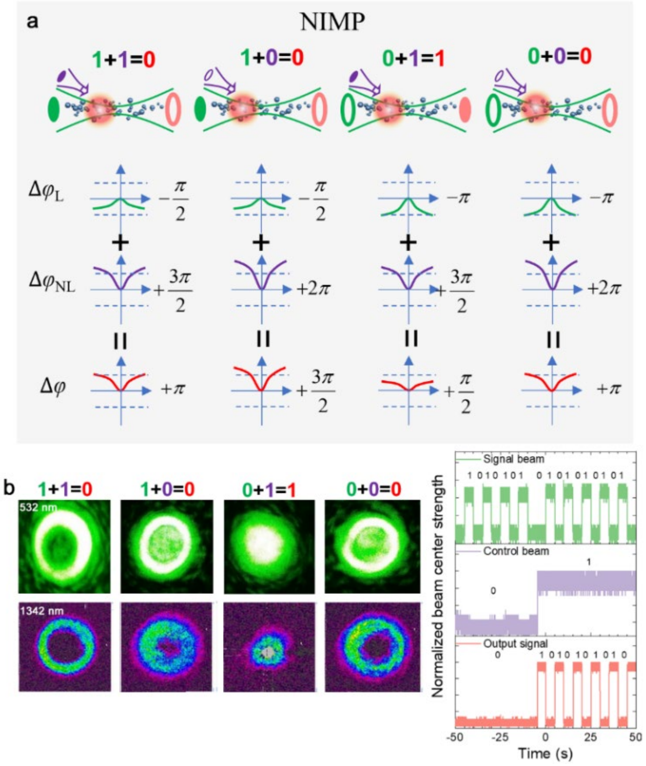

Supplementary Figure 15. Design and experimental results of ‘NIMP’ gate. (a) Mechanism of phase modulation and corresponding scheme of phase dependence. (b) Left: spatial output patterns of NIMP gate across visible and infrared region. Right: temporal input and output in processing.

Table S2. Intensity contrast and SNR of the nine logic gates

Functionality	Central intensity contrast between ‘1’ and ‘0’ patterns	SNR of output signal
AND	8:1-17:1	4.8:1
OR	2:1-10:1	21.1:1

NOT	8:1-10:1	5.1:1
NAND	10:1-16:1	31.2:1
NOR	5:1-9:1	4.1:1
XNOR	5:1-16:1	7.2:1
XOR	5:1-17:1	23.1:1
IMP	8:1-16:1	6.4:1
NIMP	8:1-17:1	6.1:1

Table S3. Main conditions of the nine logic gates

Functionality	Intensity of control beam (W/cm^2)	Distance between RIF and focal point of signal beam (mm)
AND	<1.2 $(1 + 1 = 1)$<br>4.0 $(1 + 0 = 0)$<br><1.2 $(0 + 1 = 0)$<br>4.0 $(0 + 0 = 0)$	-2.5
OR	4.0 $(1 + 1 = 1)$<br>2.2 $(1 + 0 = 1)$<br>4.0 $(0 + 1 = 1)$<br>2.2 $(0 + 0 = 0)$	2.9
NOT	11.8 $(1 \rightarrow 0)$<br>11.8 $(0 \rightarrow 1)$	3.2
NAND	11.8 $(1 + 1 = 0)$<br>7.9 $(1 + 0 = 1)$<br>11.8 $(0 + 1 = 1)$<br>7.9 $(0 + 0 = 1)$	2.6
NOR	15.9 $(1 + 1 = 0)$<br>11.8 $(1 + 0 = 0)$<br>15.9 $(0 + 1 = 0)$<br>11.8 $(0 + 0 = 1)$	2.8
XNOR	4.0 $(1 + 1 = 1)$<br>11.8 $(1 + 0 = 0)$<br>4.0 $(0 + 1 = 0)$<br>11.8 $(0 + 0 = 1)$	4.1
XOR	11.8 $(1 + 1 = 0)$<br>2.0 $(1 + 0 = 1)$<br>11.8 $(0 + 1 = 1)$<br>2.0 $(0 + 0 = 0)$	3.4
IMP	4.0 $(1 + 1 = 1)$<br>7.9 $(1 + 0 = 1)$<br>4.0 $(0 + 1 = 0)$<br>7.9 $(0 + 0 = 1)$	3.8
NIMP	11.8 $(1 + 1 = 0)$<br>15.9 $(1 + 0 = 0)$<br>11.8 $(0 + 1 = 1)$<br>15.9 $(0 + 0 = 0)$	3.1

Table S4. Comparison of Galinstan AOLG with representative optical logic-gate schemes

Platform	Functionality	Wavelength (nm)	Ref.
Gold–graphene–gold heterostructure	NOR/AND/OR/NAND	827	14
Semiconductor nanowire	NAND	458	15
Semiconductor nanowire	AND/OR/NAND	658	16
Polariton in Semiconductor microcavity	AND/OR	—	17
Ag nanowire	NOR/OR/NOT	633	18
Phase-only linear filtering for linear interference	NOT/XNOR	1542-1552	19
Perovskite photodetector	AND/OR/NAND/NOR/ NOT	400-1000	20
Rb atoms	NOT	479, 780	21
Graphene	NOR/OR/AND	1560–2100 nm	22
Semiconductor nanowire	AND/OR/NAND/NOR	532-890	23
Bulk silica and monolayer MoS_2	AND/OR/XOR/XNOR/ NOR/NAND	451-1036	24
Liquid crystal	AND/OR/NOT	532	25
Plasmonic gratings for linear interference	AND/OR/NOT/NAND/ NOR/XOR/XNOR	600-700	26
Metal structure	AND/OR/NOT/NAND/ NOR/XNOR	700-930	27
Organic nanosphere	OR	405, 350-390, 400-440	28
Black arsenic–phosphorus	OR	532, 671	29
Semiconductor optical amplifier	AND/OR/NOT/NOR/X NOR	1549.3-1557.3	30
Polariton in Semiconductor microcavity	XNOR	—	31
Boron nanosheets	OR	457-671	32
$MoSe_2$ nanoflakes	OR	405-671	33
Semiconductor optical amplifier	XOR/NOR/OR/NAND	1531.05-1553.79	34
Nonlinear fiber	AND/NOR/XOR/XNOR	1527.2-1565.6	35
All-fibre phase filters for linear interference	NOT/XNOR	1550	36
Liquid-metal nanodroplet	AND/OR/NOT/NOR/N AND/XNOR/XOR/IMP/ NIMP	532-2000 (400-4000 in potential)	This work

S. VI Possibility of nanoscale modulation via a few GNAs

The signal evolution of nanoscale SXPM modulation based on a few GNAs in free space was simulated as shown in **Supplementary Fig. 16**. The basic parameters for the simulation are that the thickness of a few GNAs is 400 nm, the wavelength of signal and control beams is 532 nm, $n_0 = 1.47$ and $n_2 = 1.68*10^{-5}$ cm^2/W. It clearly demonstrates that the nonlinear refractive index field within nanoscale distribution induced by highly confined control beam can enable efficient modulation on the spatial mode of signal beam. Such a modulation strongly depends on the change of refractive index, local range of nonlinear refractive index field, and distance of focus points between control and signal beams. As shown in **Supplementary Fig. 16a**, the signal beam diverges remarkably as the refractive-index-change increases, so that an optimal modulation depth of refractive index is proper in consideration of the power consumption of control beam. The spatial distribution range of the refractive index field is also important (**Supplementary Fig. 16b**). The modulation of signal beam is weak when the range of refractive index modulation is too small or too large. When the radius is similar with that of signal beam waist, the modulation of signal beam is modulated strongly. It means that proper focusing is important. Besides, when the position of refractive index distribution is changed, the transmitted light remarkably changes as implied by **Supplementary Fig. 16c**. The whole parameters for these simulations are listed in Table S5. These results suggest the feasibility of the proposed AOLG scheme on miniaturized integration. In addition, please note that the intensities

of signal and control beams in the simulation are around the order of 10 kW/cm², much higher than that of the experimental parameter with 1-mm cuvette. That is because the tightly focused beam in the simulation has a much short Rayleigh length that leads to a high intensity threshold (~10 kW/cm²) for '0'-'1' signal mode transition. Larger waist radius with longer Rayleigh length can lower the intensity threshold.

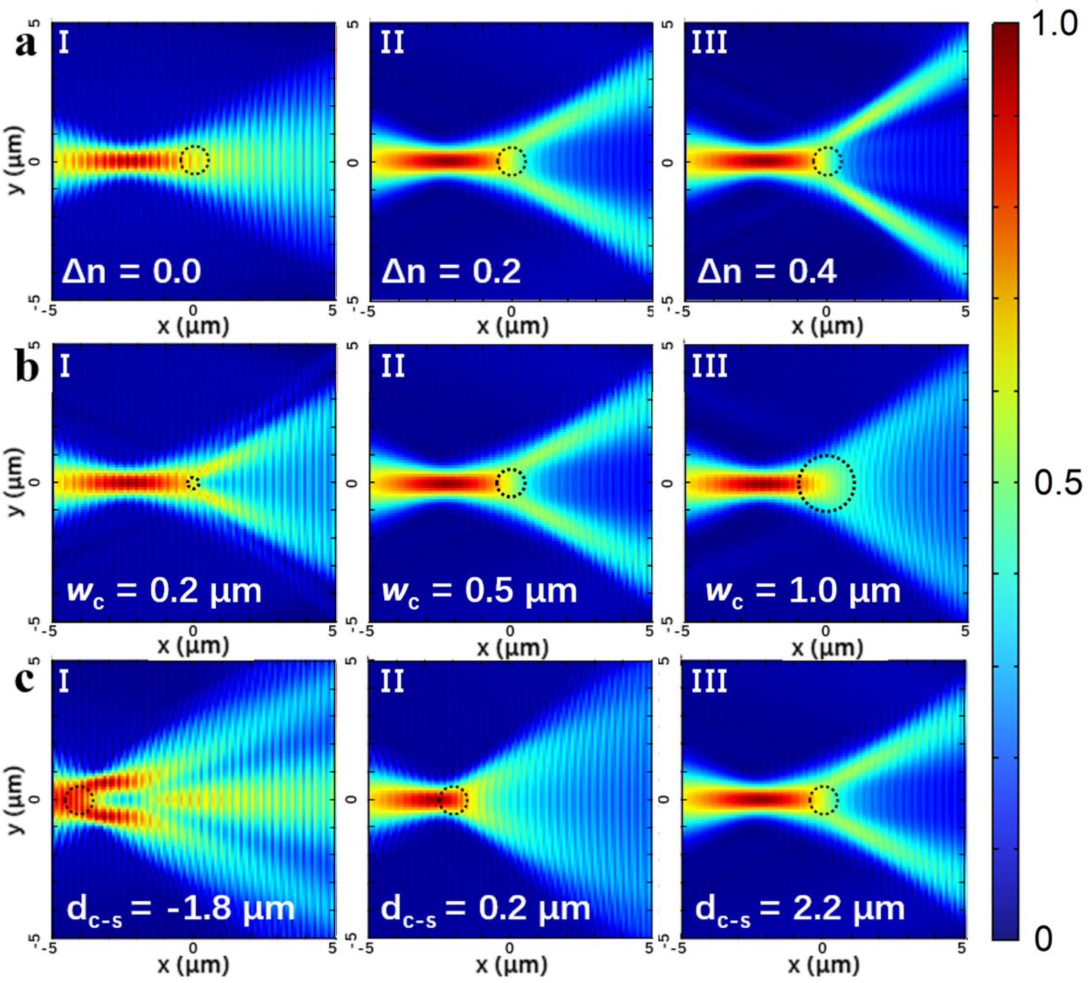

Supplementary Figure 16. Simulation of signal-beam evolution during the nanoscale all-optical SXPM modulation based on a few GNAs. (a) At different modulation depths of the refractive index. (b) At different local ranges of refractive index field. (c) At different positions of the refractive index field. d) Schematic of waveguide-based AOLG device. Black dotted circle: local range of refractive index field. Δn: change of refractive index at the waist of control beam; w_c: waist radius of control beam; d_{c-s}: distance of focus points between control and signal beams.

Table S5. Simulation parameters of Supplementary Figure 16

Figure	Control beam	Signal beam	System
a(I)	w_c = 500 nm<br>I_c = 0 W/cm^2<br>d_{c-s} = 2.2 μm	w_s = 500 nm<br>I_s < 10 kW/cm^2	Wavelength: 532 nm<br><br>Thickness of few GNAs: 400 nm<br><br>n_0 = 1.47<br>n_2 = 1.68*10^{-5} cm^2/W
a(II)	500 nm<br>12 kW/cm^2<br>2.2 μm		
a(III)	500 nm<br>24 kW/cm^2<br>2.2 μm		
b(I)	200 nm<br>12 kW/cm^2<br>2.2 μm		
b(II)	500 nm<br>12 kW/cm^2<br>2.2 μm		
b(III)	1000 nm<br>12 kW/cm^2<br>2.2 μm		
c(I)	500 nm<br>12 kW/cm^2<br>-1.8 μm		
c(II)	500 nm<br>12 kW/cm^2<br>0.2 μm		
c(III)	500 nm<br>12 kW/cm^2<br>2.2 μm		

w_c: waist radius of control beam; I_c: intensity of control beam; d_{c-s}: distance of focus points between control and signal beams; w_s: waist radius of signal beam; I_s: intensity of signal beam; n_0: linear refractive index; n_2: effective-nonlinear refractive index

S. VII References

1. Wang, G. et al. Tunable effective nonlinear refractive index of graphene dispersions during the distortion of spatial self-phase modulation. *Appl. Phys. Lett.* **104**, 141909 (2014).

2. Wang, G. et al. Tunable nonlinear refractive index of two-dimensional MoS_2, WS_2, and $MoSe_2$ nanosheet dispersions. *Photonics Res.* **3**, A51-A55 (2015).
3. Zhang, J. et al. Broadband spatial self-phase modulation of black phosphorous. *Opt. Lett.* **41**, 1704-1707 (2016).
4. Wu, L. et al. Few-Layer Tin Sulfide: A Promising Black-Phosphorus-Analogue 2D Material with Exceptionally Large Nonlinear Optical Response, High Stability, and Applications in All-Optical Switching and Wavelength Conversion. *Adv. Opt. Mater.* **6**, 1700985 (2017).
5. Lu, L. et al. Few-layer Bismuthene: Sonochemical Exfoliation, Nonlinear Optics and Applications for Ultrafast Photonics with Enhanced Stability. *Laser Photon. Rev.* **12**, 1700221 (2017).
6. Lu, L. et al. Broadband Nonlinear Optical Response in Few-Layer Antimonene and Antimonene Quantum Dots: A Promising Optical Kerr Media with Enhanced Stability. *Adv. Opt. Mater.* **5**, 1700301 (2017).
7. Wu, L. et al. Perovskite $CsPbX_3$: A Promising Nonlinear Optical Material and Its Applications for Ambient All-Optical Switching with Enhanced Stability. *Adv. Opt. Mater.* **6**, 1800400 (2018).
8. Li, X. et al. Tri-phase all-optical switching and broadband nonlinear optical response in Bi2Se3 nanosheets. *Opt. Express* **25**, 18346-18354 (2017).
9. Shi, B. et al. Broadband ultrafast spatial self-phase modulation for topological insulator Bi2Te3 dispersions. *Appl. Phys. Lett.* **107**, 151101 (2015).
10. Wu, L. et al. MXene-Based Nonlinear Optical Information Converter for All-Optical Modulator and Switcher. *Laser Photon. Rev.* **12**, 1800215 (2018).
11. Shan, Y. et al. A promising nonlinear optical material and its applications for all-optical switching and information converters based on the spatial self-phase modulation (SSPM) effect of TaSe2 nanosheets. *J. Mater. Chem. C* **7**, 3811-3816 (2019).
12. Shan, Y. et al. Spatial self-phase modulation and all-optical switching of graphene oxide dispersions. *J. Alloys Compd.* **771**, 900-904 (2019).
13. Yang, X. et al. The study of self-diffraction of mercury dithizonate in polymer film. *Opt. Commun.* **256**, 414-421 (2005).
14. Boolakee, T. et al. Light-field control of real and virtual charge carriers *Nature* **605**, 251-255 (2022).
15. Piccione, B., Cho, C. -H., Vugt, L. K. V. & Agarwal, R. All-optical active switching in individual semiconductor nanowires. *Nat. Nanotechnol.* **7**, 640-645 (2012).
16. Kim, J. et al. Photon-triggered nanowire transistors. *Nat. Nanotechnol.* **12**, 963-968 (2017).
17. Zasedatelev, A. V. et al. A room-temperature organic polariton transistor. *Nat. Photon.* **13**, 378-383 (2019).
18. Wei, H., Wang, Z., Tian, X., Käll, M. & Xu, H. Cascaded logic gates in nanophotonic plasmon networks. *Nat. Commun.* **2**, 387 (2011).
19. Maram, R. et al. Frequency-domain ultrafast passive logic: NOT and XNOR gates. *Nat. Commun.* **11**, 5839 (2020).
20. Kim, W. et al. Perovskite multifunctional logic gates via bipolar photoresponse of single photodetector. *Nat. Commun.* **13**, 720 (2022).
21. Shi, S. et al. High-fidelity photonic quantum logic gate based on near-optimal Rydberg single-photon source. *Nat. Commun.* **13**, 4454 (2022).
22. Li, Y. et al. Nonlinear co-generation of graphene plasmons for optoelectronic logic operations. *Nat. Commun.* **13**, 3138 (2022).

23. Yang, H. et al. Nanowire network−based multifunctional all-optical logic gates. *Sci. Adv.* **4**, eaar7954 (2018).
24. Zhang, Y. et al. Chirality logic gates. *Sci. Adv.* **8**, eabq8246 (2022).
25. Wang, C. -Y. et al. All-optical transistor- and diode-action and logic gates based on anisotropic nonlinear responsive liquid crystal. *Sci. Rep.* **6**, 30873 (2016).
26. Sang, Y. et al. Broadband multifunctional plasmonic logic gates. *Adv. Opt. Mater.* **6**, 1701368 (2018).
27. Yang, Z., Fu, Y., Yang, J., Hu, C. & Zhang, J. Spin-encoded subwavelength all-optical logic gates based on single-element optical slot nanoantennas. *Nanoscale* **10**, 4523-4527 (2018).
28. Hendra. et al. Photochemically switchable interconnected microcavities for all-organic optical logic gate. *Adv. Funct. Mater.* **31**, 2103685 (2021).
29. Wu, L. et al. All-optical logic devices based on black arsenic–phosphorus with strong nonlinear optical response and high stability. *Opto-Electron. Adv.* **5**, 200046 (2022).
30. Dong, J., Zhang, X., Wang, Y., Xu, J. & Huang, D. 40 Gbit/s reconfigurable photonic logic gates based on various nonlinearities in single SOA. *Electron. Lett.* **43**, 884-886 (2007).
31. Leyder, C. et al. Interference of coherent polariton beams in microcavities: polarization-controlled optical gates. *Phys. Rev. Lett.* **99**, 196402 (2007).
32. Song, C., Liao, Y., Xiang, Y. & Dai, X. Liquid phase exfoliated boron nanosheets for all-optical modulation and logic gates. *Sci. Bull.* **65**, 1030-1038 (2020).
33. Sk, K. et al. Nonlinear Coherent light-matter interaction in 2D $MoSe_2$ nanoflakes for all-optical switching and logic applications. *Adv. Opt. Mater.* **10**, 2200791 (2022).
34. Kim, J. -Y., Kang, J. -M., Kim, T. -Y. & Han, S. -K. All-optical multiple logic gates with XOR, NOR, OR, and NAND functions using parallel SOA-MZI structures: theory and experiment. *J. Lightwave Technol.* **24**, 3392-3399 (2006).
35. Li, L. et al. Reconfigurable all-optical logic gate using four-wave mixing (FWM) in HNLF for NRZ-PolSK signal. *Opt. Commun.* **283**, 3608-3612 (2010).
36. Kaushal, S. et al. All-fibre phase filters with 1-GHz resolution for high-speed passive optical logic processing. *Nat. Commun.* **14**, 1808 (2023).
